# Effects of Zofenopril on Arterial Stiffness in Hypertension Patients

**DOI:** 10.1007/s12033-023-00861-5

**Published:** 2023-09-13

**Authors:** Benjamin Palić, Ivica Brizić, Emina Karahmet Sher, Ivona Cvetković, Amina Džidić-Krivić, Heba Taha Mohmmed Abdelghani, Farooq Sher

**Affiliations:** 1 Department of Internal Medicine, University Clinical Hospital Mostar, 88000 Mostar, Bosnia and Herzegovina; 2 Department of Biosciences, School of Science and Technology, Nottingham Trent University, Nottingham, NG11 8NS UK; 3 Department of Laboratory Diagnostics, University Clinical Hospital Mostar, 88000 Mostar, Bosnia and Herzegovina; 4 Department of Neurology, Cantonal Hospital Zenica, 72000 Zenica, Bosnia and Herzegovina; 5 International Society of Engineering Science and Technology, Nottingham, UK; 6 Department of Physiology of Physical Activity, College of Sport Sciences and Physical Activity, King Saud University, 11451 Riyadh, Saudi Arabia; 7 Department of Engineering, School of Science and Technology, Nottingham Trent University, Nottingham, NG11 8NS UK

**Keywords:** Hypertension, ACE inhibitors, Sulfhydryl group, Zofenopril, Vascular Stiffness, Lipoproteins

## Abstract

Angiotensin-converting enzyme inhibitors (ACEIs) reduce arterial stiffness beyond their antihypertensive effect. Studies showed that sulfhydryl ACEIs have the antioxidative potential to improve endothelial function, which might have a clinical effect on arterial distensibility. However, there are no studies that directly compare the effects of sulfhydryl (zofenopril) and non-sulfhydryl ACEIs (enalapril) on arterial stiffness. Therefore, this prospective study aims to compare the effects of enalapril and zofenopril on arterial stiffness and oxidative stress in both short- and long-term treatment of arterial hypertension (AH). Baseline and post-treatment peripheral and central arterial pressure indices, augmentation index (Aix), aortic pulse wave velocity (ao-PWV), serum levels of oxidized low-density cholesterol lipoprotein, LDL and uric acid (UA) were measured. The results showed that acute treatment with zofenopril, in contrast to enalapril, significantly decreased peripheral and central Aix (p < 0.001). Chronic treatment with zofenopril showed a superior effect over enalapril on the reduction of the peripheral systolic arterial pressure with reduction of ao-PWV (p = 0.004), as well as a reduction in peripheral Aix (p = 0.021) and central Aix (p = 0.021). Therefore, this study indicates that zofenopril has beneficial effects on the reduction of arterial stiffness compared to enalapril. It has potent clinical efficacy in AH treatment and further studies should compare its safety and long-term efficacy to other AH drugs that would aid clinicians in treating AH and other various cardiovascular diseases that have arterial stiffness as a common denominator.

## Introduction

Arterial hypertension (AH) is one of the most commonly diagnosed diseases in Western societies, as well as in developing low-income and middle-income countries, that has an enormous burden on the healthcare system. It continues to be, in addition to other traditional risk factors, the most frequent etiological risk factor for the onset of cardiovascular diseases (CVD), such as coronary disease, myocardial infarction, heart failure, stroke, chronic renal disease, and peripheral artery disease. AH is a multifactorial disease that has been studied for decades, but there are still many unanswered questions regarding the complex underlying mechanisms leading to its onset. One of the recently discovered phenomena that is associated with AH is arterial stiffness. Arterial stiffness implies decreased distensibility of large arteries during systolic pressure increases during each heart cycle. In addition to the proven association of arterial stiffness and AH, it is believed that these two entities have a bidirectional relationship [1].

Reduced arterial distensibility causes isolated systolic hypertension, a common hypertensive phenotype in the elderly, by raising systolic arterial pressure (SAP), decreasing diastolic arterial pressure, and increasing pulse pressure (PP) [2]. On the other hand, the increased pressure stress on the arterial wall in AH results in vascular remodelling marked by elastin fibre degradation, increased collagen fibre synthesis, and hypertrophy of vascular smooth muscle cells in the medial arterial layer, which leads to a decreased distensibility in arterial stiffness. Additionally, AH induces endothelial dysfunction, with the activation of the renin-aldosterone-angiotensin-system (RAAS) [3, 4]. Along with altering vascular tone, RAAS activation induces oxidative stress by upregulating the enzyme nicotinamide adenine dinucleotide phosphate (NADPH) oxidase which increases the formation of reactive oxygen species (ROS). The involvement of the RAAS system was also found in other inflammatory health conditions, such as diabetes mellitus (DM). The effects of a hyperglycemic state and oxidative stress on the hypertrophy of arterial walls are well known [5–7].

Increased oxidative stress causes more substantial endothelial dysfunction and decreases nitric oxide bioavailability, which plays a significant pathophysiologic role in arterial stiffness, DM and AH [8, 9]. Additionally, an increase in ROS production causes low-density lipoproteins (LDL) peroxidation, resulting in the formation of oxidized LDL (ox-LDL), which is a major contributor to the development of atherosclerosis [10]. Besides ROS and ox-LDL, another molecule that has intracellular prooxidant and plasmatic antioxidant properties, is uric acid (UA) [11]. For decades, researchers have speculated that UA’s antioxidant capabilities could protect against oxidative stress, inflammation, and cell injury [12], but new epidemiological studies confirmed UA as an independent risk factor for cardiovascular disease especially AH [13]. UA induces UA-dependent AH by endothelial dysfunction and decreased NO production and it is associated with arterial stiffness, oxidative stress and inflammation [14].

It remains unclear if acute reduction of UA concentration has positive cardiovascular effects because it was proven that severe reduction of UA concentration can be potentially harmful and in AH patients produces more cardiovascular events [15, 16]. The concentration of UA in the serum depends on both its production and urine excretion. There is disagreement on how ACEIs affect urine excretion. It was discovered that ACEIs do not have a class effect on the concentration of UA, but some ACEIs have demonstrated a propensity to alter UA levels [17–20]. It is still unknown whether enalapril and zofenopril affect UA serum concentration. Arterial stiffness is a novel non-traditional risk factor for atherosclerosis and CVD. Increased pulse wave velocity (PWV), which is a gold standard for the measurement of arterial stiffness, is an independent predictor of cardiovascular mortality, myocardial infarction and stroke [21, 22].

Carotid-femoral pulse wave velocity (cf-PWV) above 10 m/s was included in the European Society of Cardiology guidelines for arterial hypertension (AH) in 2018 as a parameter of end-organ damage that could be used for risk stratification in patients with AH [23]. By using pulse oscillometry and applanation tonometry, arterial stiffness is frequently clinically and noninvasively assessed. Arteriograph (Tensiomed, Budapest, Hungary) is one of the pulse oscillometry-based instruments that is invasively verified and widely used [24]. Finding that arterial stiffness can be reduced therapeutically (arterial “de-stiffening”) using pharmacological and non-pharmacological interventions imposed an entirely novel goal in the management of AH. Numerous non-pharmacological interventions have been shown to reduce arterial stiffness, including aerobic exercise [25], calorie restriction and weight loss [26], smoking cessation [27], long-term omega-3 use [28], soy isoflavones [29] and black tea flavonoids consumption [30], decreased sodium and increased potassium intake [31] and increased intake of flavonoids [32].

The mechanisms underlying the effects of these natural compounds are still unknown, but it is most likely associated with their anti-inflammatory and antioxidant effects [33]. A variety of pharmacologic therapies, including antihypertensive drugs [34], statins [35], phosphate binder sevelamer [36], antibodies to tumour necrosis factor-alfa [37], alagebrium, an advanced glycation end-products crosslink breaker drug [38], and anti-diabetic therapy [39] have also demonstrated effectiveness in reducing arterial stiffness. Among all classes of anti-hypertensive drugs, ACEIs with angiotensin receptor blockers are proven to have the greatest impact on arterial stiffness improvement [34]. Along with reducing angiotensin II production, ACEIs also reduce arterial stiffness by a decrease of vascular remodelling, improves endothelial dysfunction by increasing the expression of endothelial nitric oxide synthase (eNOS), lowers ROS production by inhibiting NADPH oxidase and has antiatherogenic effects through reduced LDL oxidation [40, 41]. Interestingly, although these effects are mostly related to this class of antihypertensives, the structural differences between ACEIs (sulphydryl vs. non-sulphydryl) show different pharmacological and biological effects as suggested by the consensus of the working group on tissue ACEIs [42].

Previous in vitro and in vivo animal studies have shown a potential additional benefit of sulfhydryl ACEIs zofenopril and captopril in the reduction of the oxidative stress of cells in blood vessels due to the presence of sulphydryl (SH) groups that are “scavengers” of free radicals [43, 44]. Therefore, the reduction of oxidative stress in patients with AH might have a protective role on endothelial function that could further improve or slow down the development of arterial stiffness [45]. Another in vitro study noted that sulphydryl ACEI (zofenopril) is superior to non-sulphydryl ACEI (enalapril) regarding smooth muscle cells proliferation and intimal hyperplasia [46, 47]. However, further studies on humans should confirm these findings. Furthermore, research by Napoli et al. [48] demonstrated that zofenopril, as opposed to enalapril, has clinical benefits in terms of a reduction in LDL oxidability and carotid intima-media thickness, possibly resulting in favourable impacts on the prevention of atherosclerosis in patients with primary hypertension. It is still debatable if the antioxidative effects of zofenopril have clinical implications on arterial stiffness and if it is dependent on its antioxidative properties.

The phenomenon of arterial stiffness has recently been recognised as the main factor accelerating vascular ageing and the development of AH. To our knowledge, this is the first study that directly compared the effects of treatment with enalapril and zofenopril on arterial stiffness, which establishes the path for additional research on the most successful treatments for patients with AH. The primary goal of this study was to compare the effects of zofenopril (sulfhydryl ACEI) and enalapril (non-sulfhydryl ACEI) on arterial stiffness parameters during acute and chronic treatment in hypertensive patients, as well as to compare the effects of these drugs on LDL oxidability and serum LDL and UA concentration.

## Materials and Methods

### Study Design

The study is performed as a prospective study in 2 centres (University Clinical Hospital Mostar and Mostar Health Center) that consisted of two sub-studies for evaluating acute and chronic effects of enalapril and zofenopril in patients with AH. Patients with previously or newly diagnosed AH, hospitalized in the University Clinical Hospital Mostar were included in the acute effects sub-study, while outpatients with newly diagnosed AH in Mostar Health Center were part of the chronic effects sub-study. After the inclusion in the study, subjects underwent baseline evaluation and they were randomly assigned to two groups. In the first group, subjects were orally administered 20 mg of enalapril and in the second group, subjects were orally administered 30 mg of zofenopril, which were the bioequivalent doses used in previous studies [48]. The post-treatment evaluation was performed 2 h after the drugs were administered in the acute effects sub-study and 3 months after in the chronic effects sub-study. Study endpoints were changes in peripheral and central systolic blood pressure, pulse pressure, augmentation index, pulse wave velocity, serum ox-LDL and uric acid concentration. The study protocol was approved by the Ethics Committee of the University Clinical Hospital Mostar (3892/16) and the School of Medicine in Mostar (642/13). The purpose and potential risks of the study were explained to all subjects before their informed consent was obtained.

### Subjects

The sub-study of acute effects included 65 subjects hospitalized at the Clinic of Internal Medicine, with previously or newly diagnosed AH following the ESC guidelines for AH [42]. Each patient had a medical history review, physical examination, and laboratory testing to identify any potential exclusion criteria. Excluding criteria were the following conditions: acute or chronic heart failure, significant valvular disease, arrhythmias including frequent ventricular ectopy, acute infectious diseases, severe electrolyte disturbances, acute kidney injury and chronic kidney disease (eGFR < 30 mL/min/1.73m^2^), acute liver failure, gastrointestinal bleeding and food or nicotine consumption between measurements. Seven subjects were excluded from the study (4 subjects refused the second measurement, and 3 subjects consumed food after initial evaluation). A total of 58 subjects completed the study. Out of 58 enrolled subjects, 27 were in the enalapril group (68.7 ± 10.7 years; M 15) and 31 subjects were in the zofenopril group (66.6 ± 14.2; M 18).

The sub-study of chronic effects included a total of 63 patients who were newly diagnosed with AH by their general practitioner and required antihypertensive treatment. The exclusion criteria were the existence of any other chronic disease or concomitant drug therapy. Nineteen patients were excluded from the study. Nine patients had therapy changes, 5 patients started taking additional drug therapy and 5 patients discontinued therapy on their own. A total of 44 subjects completed the study and 20 included subjects were in the enalapril group (55.4 ± 10.5; M 11) and 24 subjects were in the zofenopril group (58.4 ± 14.4; M 15). All patients in both sub-studies underwent baseline and post-treatment measurements of arterial stiffness by pulse oscillometry and peripheral venous blood sampling. Patients were prohibited from food, alcohol and cigarette consumption for a minimum of 12 h before evaluation. Hospitalized patients have not been administered any medication for the last 12 h.

### Studied Drugs

Zofenopril 30 mg and enalapril 20 mg, which were used in this study, were administered orally with 200 mL of water. Both drugs have long been used as antihypertensives and have received approvals from European Medicines Agency (enalapril P/0093/2021 and zofenopril CPMP/2011/00) and U.S. Food and Drug Agency (enalapril 18-998/S059). Zofenopril ([1(S),4(S)]-1(3-mercapto-2-methyl-1-oxopropyl)4-phenylthiol-l-proline-S-benzothioester) is sulphydryl ACEI that was administered in the form of hemicalcium salt zofenopril calcium, while enalapril ((S)-1-[N-[1-(ethoxycarbonyl)-3-phenylpropyl]-l-alanyl]-l-proline) is carboxyl ACEI that was administered in the form of maleic acid salt. Both drugs are pro-drugs that are actively hydrolyzed into their active forms, zofenoprilat and enalaprilat, respectively. While maximum enalapril concentration occurs after 1 h and induces maximum ACEI after 2 h, maximum serum zofenoprilat concentration occurs 1.4 h after peroral dosing and induces total ACE inhibition up to 9.4 h. Zofenopril is mostly metabolized and eliminated by the kidney and liver, whereas enalapril is eliminated through the kidneys [49]. Zofenopril has a higher lipophilicity than enalapril, which results in deeper tissue penetration and longer-lasting tissue ACE inhibition (heart, aorta) [50]. Absorption of zofenopril after oral administration is full and there is no loss of dose due to intestine activity, compared to enalapril [51]. Additionally, zofenopril has a better safety profile in terms of dry cough incidence when compared to enalapril due to its reduced potential to induce cyclooxygenase-2 and bradykinin production [52].

### Measurement of Arterial Stiffness

Arterial stiffness was assessed noninvasively by an automated oscillometry and validated device Arteriograph (Tensiomed, Hungary, Budapest) [24, 53]. All measurements were performed by a single well-trained clinician in the supine position after a 10-min rest in a quiet, temperature-controlled environment. The brachial cuff was positioned on the dominant arm and the distance from the jugulum to the pubic symphysis was used for automatic analysis. The device inflated the brachial cuff to 35–40 mmHg above the brachial SAP and for 2 min recorded the pulsatile waveform from the brachial artery through a pressure sensor. The first systolic peak of the recorded waveform corresponds to the ejection of the left ventricle, whereas the second peak represents the reflected wave from the periphery. The difference in the time between the ejected and the reflected wave is used for PWV calculation (PWV = Jugular-pubic distance/time). The augmentation index (Aix) represents pressure augmentation over the pulse pressure. The calculation of aortic SAP is based on the relationship between invasively measured SAP in the aorta and the brachial artery, based on the late systolic wave amplitude. All subjects underwent three measurements at 5-min intervals and the average value of the last 2 measurements was used.

### Determination of MDA-OxLDL and Uric Acid

Peripheral venous blood sampling (antecubital vein) of 8–10 mL blood was performed. After plasma separation in tubes without anticoagulants, it was centrifuged 3 times (1000 RPM) at 4 °C and stored in micro sample tubes with gel (Sarstedt, Nümbrecht, Germany; Volume 1.1 mL) at − 80 °C until analysis. The ox-LDL concentration in serum was determined by enzyme-linked immunosorbent assay using a commercial ox-LDL/MDA-Adducts ELISA kit (DRG Instruments Gmbh, Germany) on EZ Read 800 Plus analyzer (Biochrom, USA) as described [54]. The concentration of uric acid was determined by spectrophotometry using a uric acid reagent kit (Beckman Coulter, Co. Clare, Ireland) on Beckman Coulter Chemistry Analyzer AU680 (Beckman Coulter, Brea, USA) [55], while the concentration of LDL was determined by spectrophotometry using LDL reagent kit (Beckman Coulter, Co. Clare, Ireland) on Beckman Coulter Chemistry Analyzer AU680 (Beckman Coulter, Brea, USA) [56].

### Statistical Analysis

Continuous data are presented as average values (± standard deviation, SD), whereas nominal data are displayed as a frequency (percentage). The Kolmogorov-Smirnov test was used to determine the normality of a sample’s data distribution. When the normal distribution was satisfied, the paired t-test was used to compare within-group effects before and after treatment, and a nonparametric Wilcoxon test was used when the normal distribution was violated. Mixed model ANOVA was used for the analysis of between-group effects (enalapril vs. zofenopril) [57]. A statistically significant difference was taken for p < 0.05. The statistical tool used for data evaluation is SPSS for Windows program, version 17.0 (SPSS Inc., Chicago, USA).

## Results and Discussion

### Acute Effects of Enalapril and Zofenopril Treatment

**Fig. 1:**
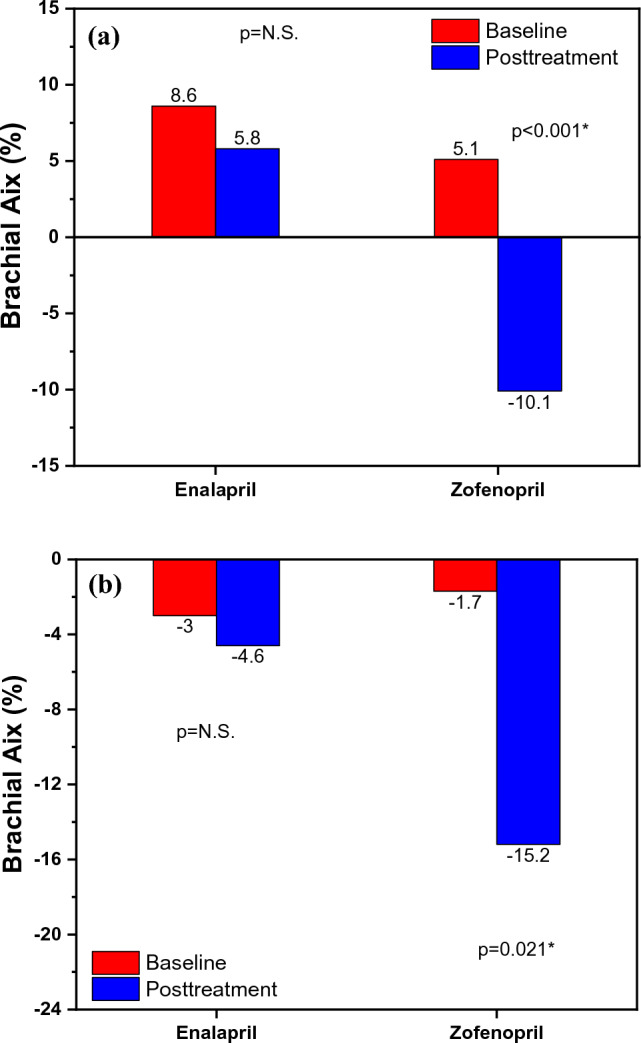
**a** Acute effects on brachial Aix and **b** Chronic effects on brachial Aix within the groups before and after the treatment with enalapril and zofenopril. Data are represented as average values, p < 0.05

**Fig. 2:**
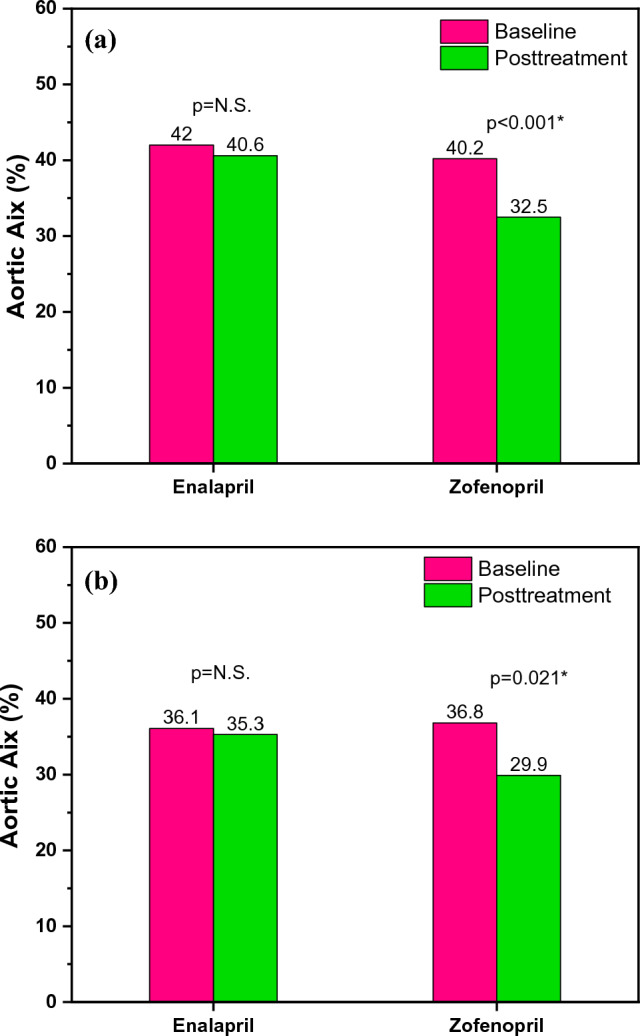
**a** Acute effects on aortic Aix and **b** Chronic effects on aortic Aix within the groups before and after the treatment with enalapril and zofenopril. Data are represented as average values, p < 0.05

**Fig. 3:**
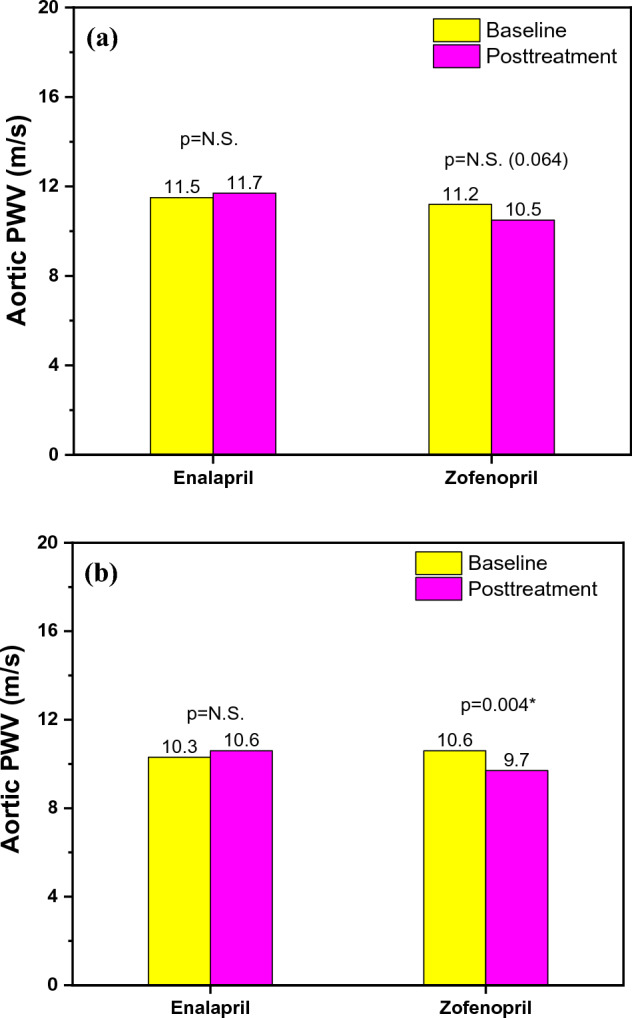
**a** Acute effects on aortic PWV and **b** Chronic effects on aortic PWV within the groups before and after the treatment with enalapril and zofenopril. Data are represented as average values, p < 0.05

Patients included in the acute effects sub-study were assessed by age, sex, body mass index (BMI), central and peripheral systolic arterial pressure (SAP) and pulse pressure (PP), as it is shown in Table 1. The patients in the enalapril group had an average age of 68.7 years, whereas those in the zofenopril group had an average age of 66.6 years. Patients in both treatment groups were mostly overweight, with an average BMI of 26. As expected, both drugs reduced central aortic SAP (enalapril 9.9 mmHg; p = 0.031 vs. zofenopril 11.9 mmHg; p < 0.001) and peripheral brachial SAP (enalapril 5.1 mmHg; p = 0.016 vs. zofenopril 8.3 mmHg; p < 0.001) with no statistical difference between groups (central SAP p = 0.29; peripheral SAP p = 0.20). Zofenopril significantly reduced brachial PP (4.1 mmHg; p = 0.001) and aortic PP (7.6 mmHg; p < 0.001) while enalapril reduced only aortic PP (2.7 mmHg; p = 0.042). These effects are shown in Table 2. Additionally, zofenopril significantly reduced brachial Aix by 15.2% (p < 0.001) (Fig. 1a) and aortic Aix by 7.7% (p < 0.001) (Fig. 2a), which was not found in the enalapril group. In contrast to zofenopril, which lowered aortic PWV by 0.7 m/s with nearly statistical significance (p = 0.064), enalapril did not have a clinically significant impact on aortic PWV, as it is shown in Fig. 3a. This study also hypothesized that zofenopril, which has free SH groups in its structure, acts as an antioxidative agent. Consequently, as a result of the enhanced NO bioavailability and subsequent improvement in endothelial function, it may reduce arterial stiffness, but it may also have superior effects on blood pressure reduction.

**Table 1:** Baseline demographic and clinical characteristics of patients between treatment groups in the acute effects sub-study

Treatment	Enalapril (N 27)		Zofenopril (N 31)	
Age (years)	68.7 ± 10.7		66.6 ± 14.2	
Sex (N, %)	M	F	M	F
	15 (55.6%)	12 (45.4%)	18 (58.1%)	13 (41.9%)
BMI	26.8 ± 4.3		26.6 ± 2.7	
Brachial SAP (mmHg)	139.4 ± 27.5		133.8 ± 16.4	
Brachial PP (mmHg)	54.9 ± 15.5		54.3 ± 10.9	
Aortic SAP (mmHg)	141.9 ± 30.5		135.6 ± 18.8	
Aortic PP (mmHg)	59.2 ± 20.7		56.1 ± 13.4	
Ox-LDL (ng/mL)	12.7 ± 11.3		19.1 ± 18.2	
LDL (mmol/L)	3.6 ± 1.4		3.2 ± 0.9	
Uric acid (µmol/L)	386 ± 125		367 ± 136	

Data are presented as average value ± SD

*BMI* body mass index; *SAP* systolic arterial pressure; *PP* pulse pressure; *ox*-*LDL* oxidized low-density lipoproteins; *LDL* low-density lipoprotein

**Table 2:** Baseline and post-treatment effects of enalapril and zofenopril on aortic and brachial SAP, PP, ox-LDL, LDL and uric acid in the acute effects sub-study

Treatment	Enalapril (N 27)		Zofenopril (N 31)		Effects between group
	Baseline	Post-treatment	Baseline	Post-treatment	(p-value)
Brachial SAP (mmHg)	139.4 ± 27.5	134.3 ± 26.3*	133.8 ± 16.4	125.5 ± 17***	0.209
Brachial PP (mmHg)	54.9 ± 15.5	54.5 ± 15.8	54.3 ± 10.9	50.2 ± 10.2**	0.461
Aortic SAP (mmHg)	141.9 ± 30.5	132 ± 37.3**	135.6 ± 18.8	123.7 ± 19.1***	0.120
Aortic PP (mmHg)	59.2 ± 20.7	56.5 ± 18*	56.1 ± 13.4	48.5 ± 12.9***	0.190
Ox-LDL (ng/mL)	12.7 ± 11.3	14 ± 13.9	19.1 ± 18.2	18.9 ± 19.7	0.428
LDL (mmol/L)	3.6 ± 1.4	3.8 ± 1.4	3.2 ± 0.9	3.3 ± 1.1	0.138
Uric acid (µmol/L)	386 ± 125	446 ± 182	367 ± 136	363 ± 134	0.287

Data are presented as average value ± SD

*SAP* systolic arterial pressure; *PP* pulse pressure; *ox*-*LDL* oxidized low-density cholesterol lipoproteins; *LDL* low-density lipoprotein

*p<0.05; **p<0.01; ***p<0.001 (paired t-test for within group effects and mixed model ANOVA for between group effects was used)

This research confirmed that both drugs significantly decreased blood pressure in acute treatment. Both drugs decreased brachial SAP, aortic SAP and aortic PP, while zofenopril additionally reduced brachial PP. Although zofenopril effects were more pronounced in terms of blood pressure (BP) reduction in acute treatment, there was no statistical significance when the statistical analysis between these two groups was conducted. Similar findings of BP reduction to this study were reported in Mallion’s study, which compared enalapril and zofenopril in terms of peripheral SAP decrease. It was found that zofenopril showed earlier (within 2 weeks) lowering of BP compared to enalapril with a difference of about 3.5 mmHg in zofenopril’s favour [58]. Shown differences in BP reduction between the two drugs used in this study, which are in favour of zofenopril (central SAP 2.0 mmHg, peripheral SAP 3.2 mmHg) but still not statistically significant, are clinically relevant because the VALUE trial noted that minimal BP reduction with amlodipine (4.0/2.1 mmHg) and valsartan (1.5/1.3 mmHg) had a significant effect in terms of myocardial infarction reduction (19%) and stroke incidence (15%) [59]. Zofenopril’s clinical effect in terms of greater central and peripheral SAP reduction, although not significant in this study, might be the consequence of increased NO bioavailability as suggested in studies, although the concentration of NO was not measured in our research and further studies should evaluate this proposition [60, 61].

Both drugs did not show a significant effect on ao-PWV, although it is crucial to note that zofenopril demonstrated nearly statistical significance with a clinically meaningful decrease in ao-PWV by 0.7 m/s. Interestingly, this result was similar to the results of a meta-analysis that noted that the short-term treatment (from 2 h to less than 4 weeks) with ACEI led to cf-PWV reduction of around 0.75 m/s [62]. Moreover, it was also demonstrated that in a shorter time (2 h after administration), quinapril, which is an ACEI inhibitor without a sulfhydryl group, significantly decreased PWV for almost 1 m/s [63]. However, the most important finding in the sub-study researching the acute effects of zofenopril was the significant reduction in central and peripheral Aix. Although the NO concentration was not measured in this study, assumption was that Aix reduction was a consequence of endothelial function improvement due to an increase in the NO bioavailability, as also suggested in the work of Napoli et al. [48].

### Chronic Effects of Enalapril and Zofenopril Treatment

Patients included in the chronic effects sub-study did not differ by age, sex, BMI and PP. Subjects in the zofenopril group had higher central and peripheral SAP. Patients in the enalapril group had an average age of 55.4 years, whereas those in the zofenopril group had an average age of 58.4 years. In both treatment groups, patients were mostly overweight, with an average BMI of 28. Table 3. displays the baseline characteristics of the treatment groups. Both drugs resulted in the reduction of central aortic SAP (enalapril 9.5 mmHg; p = 0.033 vs. zofenopril 15.5 mmHg; p = 0.001) and peripheral brachial SAP (enalapril 9.2 mmHg; p = 0.021 vs. zofenopril 12.1 mmHg; p = 0.001), with a reduction of aortic PP only in the zofenopril group (8.3 mmHg; p = 0.009). In a between-group comparison, zofenopril outperformed enalapril in the brachial SAP reduction by 2.9 mmHg (p = 0.017), as shown in Table 4. This study indicated that only chronic treatment with zofenopril exhibits considerable superiority to enalapril in terms of peripheral SAP reduction, in contrast to studies by Mallion et al. that suggested zofenopril’s acute superiority over enalapril diminished after 12 weeks of treatment. It is unknown at what time zofenopril surpasses enalapril because this study did not evaluate participants monthly and this needs to be investigated in future studies.

**Table 3:** Baseline demographic and clinical characteristics of patients between treatment groups in the chronic effects substudy

Treatment	Enalapril (N 20)		Zofenopril (N 24)	
Age (years)	55.4 ± 10.5		58.4 ± 14.4	
Sex (N, %)	M	F	M	F
	11 (55%)	9 (45%)	15 (62.5%)	9 (37.5%)
BMI	28.2 ± 4		28.1 ± 3.4	
Brachial SAP (mmHg)	145 ± 14.1		155.4 ± 16.3*	
Brachial PP (mmHg)	52.6 ± 10		59.5 ± 12.7	
Aortic SAP (mmHg)	145.7 ± 17.5		156.7 ± 22.4*	
Aortic PP (mmHg)	53.4 ± 13.6		60.8 ± 16.7	
Ox-LDL (ng/mL)	15.4 ± 13.9		9.2 ± 3.6	
LDL (mmol/L)	3.8 ± 1.2		3.6 ± 0.9	
Uric acid (µmol/L)	360 ± 113		354 ± 86	

Data are presented as average value ± SD

*BMI* body mass index; *SAP* systolic arterial pressure; *PP* pulse pressure; *ox*-*LDL* oxidized low-density lipoproteins; *LDL* low-density lipoprotein

*p<0.05

**Table 4:** Baseline and post-treatment effects of enalapril and zofenopril on aortic and brachial SAP, PP, ox-LDL, LDL and uric acid in the chronic effects substudy

Treatment	Enalapril (N 20)		Zofenopril (N 24)		Effects between group
	Baseline	Post-treatment	Baseline	Post-treatment	(p-value)
Brachial SAP (mmHg)	145 ± 14.1	135.8 ± 11.2*	155.4 ± 16.3	143.3 ± 14.6***	0.017*
Brachial PP (mmHg)	52.6 ± 10	50.1 ± 8.5	59.5 ± 12.7	55 ± 11.8	0.058
Aortic SAP (mmHg)	145.7 ± 17.5	136.2 ± 13*	156.7 ± 22.4	141.2 ± 20.2***	0.121
Aortic PP (mmHg)	53.4 ± 13.6	51.4 ± 11.6	60.8 ± 16.7	52.5 ± 15.7**	0.289
OxLDL-MDA (ng/mL)	15.4 ± 13.9	17 ± 21	9.2 ± 3.6	9.2 ± 4.3	0.302
LDL (mmol/L)	3.8 ± 1.2	3.7 ± 1.1	3.6 ± 0.9	3.6 ± 1	0.409
Uric acid (µmol/L)	360 ± 113	366 ± 100	354 ± 86	352 ± 76	0.842

Data are means ± SD

*BMI* body mass index; *SAP* systolic arterial pressure; *PP* pulse pressure; *ox*-*LDL* oxidized low-density lipoproteins; *LDL* low-density lipoprotein

*p<0.05; **p<0.01; ***p<0.001 (Wilcoxon signed-rank test for within group effects and mixed-model ANOVA for between group effects was used)

As hypothesized, chronic treatment with zofenopril after 12 weeks reduced arterial stiffness. Similar to the acute effects sub-study, zofenopril decreased brachial Aix by 13.5% (Fig. 1b) and aortic Aix by 6.9% (Fig. 2b), while enalapril did not show this effect. Additionally, compared to enalapril, chronic treatment with zofenopril resulted in a clinically significant (0.9 m/s) reduction in aortic PWV (p = 0.004) (Fig. 3b). Finally, the comparison of the findings of the sub-study regarding acute effects of zofenopril highlighted that only long-term zofenopril treatment reduces arterial stiffness in terms of ao-PWV reduction. After a review of the literature, the ZEUS study was the only research that evaluated the effects of zofenopril on arterial stiffness [64]. According to the ZEUS research, 18 weeks of treatment with zofenopril/hydrochlorothiazide lowered PWV by 0.8 m/s and Aix by 4.4%, which is similar to the findings obtained from our study. The aforementioned meta-analysis, which included 15 RCT studies, showed that the long-term ACEI treatment (longer than a month), compared to other classes of antihypertensive drugs, led to a reduction of cf-PWV for 1.5 m/s, noted that only quinapril was used in the ACEI group of drugs and further research should include other ACEI and compare their effects in this class of antihypertensives [62]. Despite equal BP reduction, a reduction in ao-PWV in chronic zofenopril treatment but not acute zofenopril treatment indicates that zofenopril improves arterial stiffness in a time-dependent manner because of the various changes noted in the structural characteristics of the artery wall.

Zofenopril’s effects on vascular remodelling were already established in a mouse model of carotid artery stenosis, where it was demonstrated that after a one-month treatment period, zofenopril reduced intimal hyperplasia and vascular smooth muscle cell proliferation. These outcomes were not found in the mice groups treated with enalapril [47, 65]. Another hypothesis for the explanation of zofenopril’s chronic effects, as previously suggested by Momose et al., is that the increased availability of NO downregulates endothelin-1 gene expression. In specific, the expression of this gene regulates the production of endothelin-1 (ET-1), a peptide that is crucial in the regulation of the vasomotor tone and the balance between NO and endothelin-1 [66]. In the work of Desideri et al. [67], it was confirmed that sulphydryl ACEI (zofenopril) significantly reduced ET-1 production when compared to the non-sulphydryl ACEI. Dillon et al. [68] also examined the effects of ACEI with the sulfhydryl group (captopril) on microvascular vasodilation. It was confirmed, by the use of laser-Doppler flowmetry, that sulphydryl ACEI following 16 weeks of therapy improved cutaneous microvascular endothelium-dependent vasodilation in patients with AH, in part via hydrogen sulphide-dependent mechanisms [66]. The significant reduction of ao-PWV in the zofenopril group was the most notable discovery in the chronic effects sub-study, most likely due to time-dependent structural changes in the arterial wall and the aforementioned bio humoral adaptations that are responsible for the vasomotor tone of the blood vessels.

### Acute and Chronic Effects of Enalapril and Zofenopril Treatment on ox-LDL, LDL and Uric acid

**Fig. 4:**
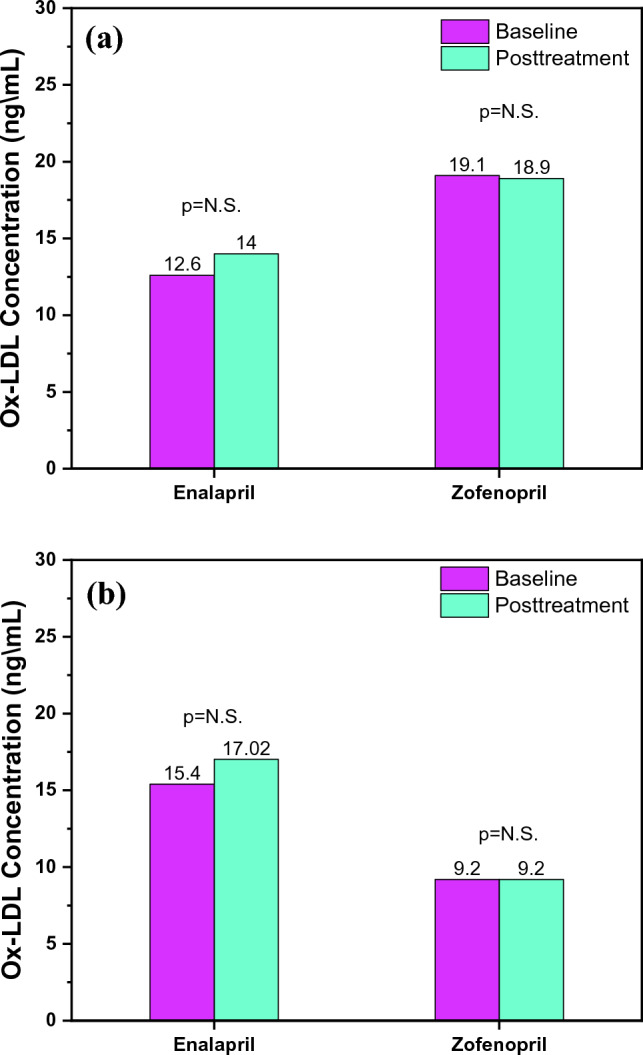
**a** Acute effects on serum ox-LDL concentration and **b** Chronic effects on serum ox-LDL concetration within the groups before and after the treatment with enalapril and zofenopril. Data are represented as average values, p < 0.05

This study did not find any differences in the reduction of circulating levels of ox-LDL between the usage of either of these drugs, both in the acute and the chronic treatment, as shown in Fig. 4a–b. This is in contrast to previous in vitro and in vivo studies, as well as other studies that were conducted on hypertensive patients. The study by Pasini et al. [69] compared the effects of ramipril (non-sulphydryl ACEI), atenolol (beta blocker) and zofenopril (sulphydryl ACEI) on the reduction of LDL hydroperoxide, circulating ox-LDL and plasma-8 isoprostane in patients with AH. Interestingly, the findings obtained in this study revealed that non-sulphydryl ACEI had no effect whereas zofenopril significantly reduced plasma-8 isoprostane, circulating ox-LDL, and LDL hydroperoxide. Another study by Napoli et al. [48] also examined the effects of enalapril and zofenopril on the reduction of the levels of oxidative molecules in individuals with essential arterial hypertension. It has been demonstrated that zofenopril has a more potent ability to decrease the levels of oxidative markers, such as malondialdehyde and isoprostane, with an increase in plasmatic NO levels.

**Fig. 5:**
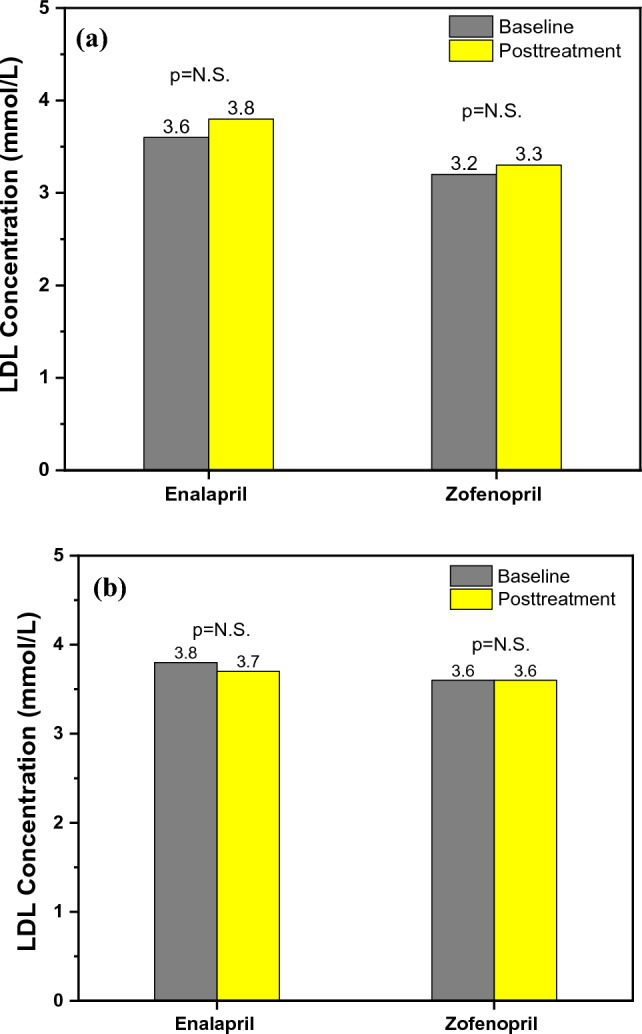
**a** Acute effects on serum LDL concentration and **b** Chronic effects on serum LDL concentration within the groups before and after the treatment with enalapril and zofenopril. Data are represented as average values, p < 0.05

One of the limitations of our study, as well as a potential explanation for why zofenopril’s antioxidant properties could not be demonstrated, was the use of only one indirect measure of oxidative stress (serum ox-LDL concentration). This could have been affected by the unique metabolism of ox-LDL due to clearance by the reticuloendothelial system, by the activation of the LOX-1 receptor on endothelial cells, as well as by the ox-LDL autoantibodies [10]. The serum LDL levels in our study did not alter in either the acute or chronic sub-studies, as shown in Fig. 5a–b. Regarding the impact of ACEIs on blood lipid status, there are conflicting results. Enalapril, lisinopril, and perindopril were all used in the study by Oksa et al. on 52 patients with AH. It was noted that they did not affect lipid status [70]. Furthermore, Krysiak et al. found that enalapril and perindopril did not affect lipid status in patients with coronary artery disease [71]. Another study also supported that individuals with essential hypertension, who used captopril (sulphydryl ACEI), had lower levels of total cholesterol, LDL cholesterol, and triglycerides [72]. The results of our research contribute to a better understanding of the way ACEIs affect lipid status. However, larger interventional randomized controlled trials are required to further investigate these effects.

**Fig. 6:**
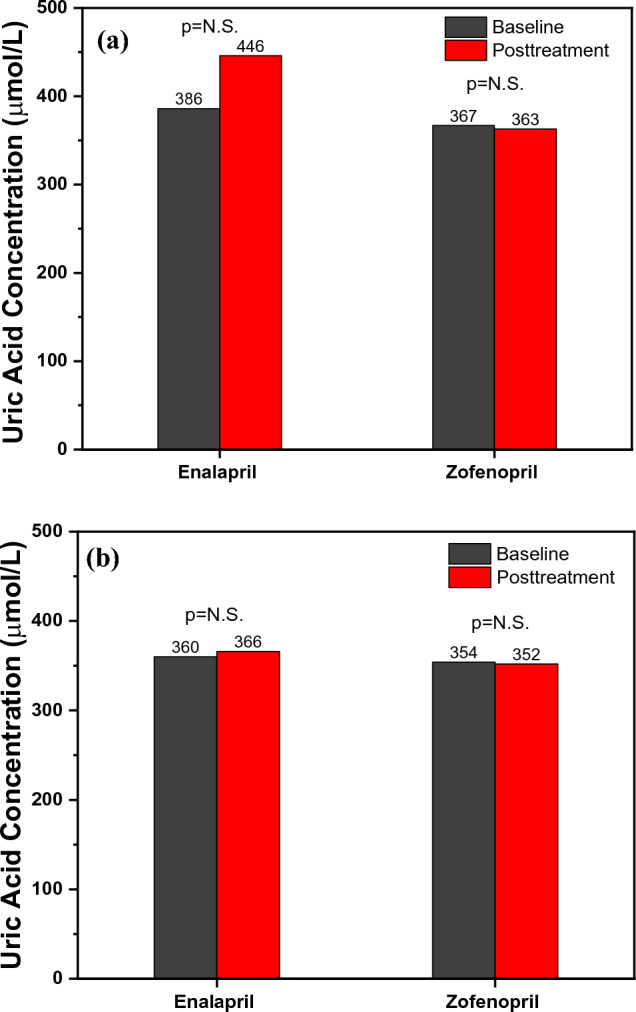
**a** Acute effects on serum UA concetration and **b** Chronic effects on serum UA concentration within the groups before and after the treatment with enalapril and zofenopril. Data are represented as average values, p < 0.05

Serum UA concentration increased after the acute enalapril treatment, but it was not statistically significant, as shown in Fig. 6a. In contrast, zofenopril acute treatment had no such effects. Chronic treatment in both treatment groups did not show any effects on serum UA concentration, as seen in Fig. 6b. As was already mentioned, numerous investigations have found conflicting results about ACEIs effects on serum UA. According to Kim et al. [18] study, ARB does lower UA, but ACEIs only lower UA in individuals with glomerular filtration rates < 60 mL/min/1.73 m^2^ [18]. A similar increase in serum UA following 24 months of enalapril therapy was found in the study of Bryant et al. [19], while acute treatment with ramipril induced UA serum decrease 7 h after treatment [17]. After a literature review, we did not find any studies regarding zofenopril’s effect on serum UA concentration. The finding that the increase in serum UA after acute enalapril therapy did not result in an expected increase in brachial and aortic Aix challenges the hypothesis that UA exerts a negative impact on endothelial function. For the evaluation of these effects in future research, more studies utilizing endothelial function testing are required.

There are several limitations regarding this study. Firstly, the main limitation was the small sample size of patients, with an increased possibility for selection bias to occur. Another limitation of this study is that it was conducted at just one larger clinical hospital. Therefore, a study carried out in a large multicentre clinical trial is required for some broad conclusions on this topic and the confirmation of our obtained data. Furthermore, it was demonstrated that zofenopril improved ao-PWV after 12 weeks of treatment, however, arterial stiffness assessment during that period was not undertaken. To check for a potential earlier change in arterial stiffness, future studies should include one-month time points for the measurement of ao-PWV. The use of only one parameter in the assessment of antioxidative effects was another potential issue. To obtain more accurate results, it would be necessary to assess several antioxidative markers, including LDL-hydroperoxide, MDA, and isoprostane. Additionally, the NO level in the serum should be correlated with these findings, and flow-mediated dilation should be considered when assessing endothelial function.

## Conclusion

This study showed that zofenopril improved arterial stiffness as well as led to a reduction of aoPWV without an effect on circulating levels of ox-LDL in patients with AH. The main conclusion is summarised as follows:

- Enalapril and zofenopril both reduced blood pressure in acute and chronic settings, while zofenopril showed a superior effect compared to enalapril in terms of brachial systolic pressure reduction in chronic treatment.
- Zofenopril showed a reduction of Aix in acute settings (An indirect marker of arterial stiffness) with a reduction of ao-PWV (A direct marker of arterial stiffness) in chronic treatment that was not found in the enalapril group. These outcomes were independent of serum ox-LDL, LDL and UA concentration, which was unaffected by either medication in either setting.
- Zofenopril’s superiority as a sulphydryl ACEI may play a crucial role in future antihypertensive treatment guidelines, given the anticipated decrease in cardiovascular mortality and morbidity after the reduction of arterial stiffness. To confirm this and to follow up on zofenopril’s long-term effects on the overall mortality of patients with AH, larger trials need to be conducted.
- Zofenopril’s effects on endothelial function should be evaluated further to investigate whether it affects NO concentration and clinically by flow-mediated dilation.
